# Apigenin promotes remodeling of peripheral and skeletal adipocytes in response to β3-AR and TLR4 activation

**DOI:** 10.3389/fendo.2025.1633584

**Published:** 2025-10-17

**Authors:** Meshail Okla

**Affiliations:** ^1^ Department of Community Health Sciences, College of Applied Medical Sciences, King Saud University, Riyadh, Saudi Arabia; ^2^ Stem Cell Unit, Department of Anatomy, College of Medicine, King Saud University, Riyadh, Saudi Arabia

**Keywords:** Apigenin, adipocyte browning, inflammation, toll-like receptor 4 (TLR4), adaptive thermogenesis

## Abstract

**Background:**

Inflammation impairs adipocyte browning and diminishes the protective role of adaptive thermogenesis against obesity and metabolic disorders. The aim of this study is to evaluate the effects of the anti-inflammatory dietary compound apigenin (Api) on adaptive thermogenesis in C57BL/6 mice in the presence of inflammation and to determine whether changes occur in skeletal adipocytes in these mice.

**Methods:**

Lipopolysaccharide (LPS) was administered intraperitoneally at 8 µg/mouse every other day for 2 weeks to activate TLR4 signaling. Api was administered intraperitoneally at 30 mg/kg every other day for 2 weeks. To stimulate thermogenesis, CL316,243 (CL), a β3-adrenergic receptor agonist, was administered at 1 mg/kg during the final 5 days. Accordingly, four experimental groups were created: control, CL, LPS+CL, and Api+LPS+CL.

**Results:**

Our results show that Api restored CL-induced thermogenesis in LPS-injected mice. This effect was mediated by inguinal white adipose tissue (iWAT) browning, accompanied by reduced inflammation and alterations in genes involved in fatty acid oxidation and *de novo* lipogenesis. The thermogenic effect of Api was specific to iWAT and was not observed in brown adipose tissue or visceral fat. Notably, these metabolic changes were associated with bone marrow fat expansion, without affecting osteogenic markers or trabecular and cortical bone parameters.

**Conclusions:**

Collectively, Api positively regulates thermogenesis and lipid metabolism in iWAT of LPS-injected mice and promotes lipid accumulation in the bone marrow without causing perturbations in bone parameters. Therefore, Api holds promise for restoring thermogenesis under inflammatory conditions. However, further investigation is needed to elucidate the impact of Api-induced adipose tissue remodeling on skeletal health, particularly under conditions of prolonged supplementation.

## 1 Introduction

Obesity-related dysfunctions in adaptive thermogenesis have been attributed to inflammatory pathways in several reports (1, 2). Notably, TNF-α plays a significant role in β3-adrenoreceptor deficiency in cultured brown adipocytes, while the absence of TNF receptors reduces brown adipocyte apoptosis and enhances β3-adrenoreceptor and UCP-1 expression in obese mice (3). In addition, TLR4 activation by either a high-fat diet (HFD) or LPS impairs adaptive thermogenesis in mice, whereas deletion of TLR4 protects adipose tissue browning in the presence of inflammation (4). Macrophages can also interact with adipocytes and interfere with their browning potential (5, 6). For instance, direct adhesive interactions between macrophages and adipocytes via the α4 integrin-VCAM-1 interaction have been reported to inhibit beige adipogenesis during obesity (7). Additionally, activated macrophages and their secreted proinflammatory cytokines have been shown to interfere with UCP1 induction in white adipocytes (6, 8). Specifically, macrophage-derived IL-1β has been identified as a key cytokine that represses the induction of UCP1 in adipocytes (6, 9). However, other cytokines elevated in obesity, such as IL-18 and IL-10, play distinct roles in brown adipose tissue (BAT) thermogenesis. For example, IL-18 has been reported to be essential for BAT thermogenesis (10). In contrast, adipocyte thermogenesis and energy expenditure were induced in mice in the absence of the anti-inflammatory cytokine IL-10 (11). Therefore, the link between inflammation and adaptive thermogenesis is complex, and its molecular basis is not clearly understood.

The natural product apigenin (Api; 4’,5,7-trihydroxyflavone), a member of the flavone family of flavonoid compounds found in many fruits and leafy vegetables, offers desirable health benefits (12, 13). Previous studies have shown that Api provides protection against obesity and related metabolic disturbances (14–16). Api prevented the progression of NAFLD in HFD-fed mice by regulating lipid metabolism and oxidative stress through mechanisms associated with Nrf2 activation (14). Additionally, long-term supplementation with a low dose of Api ameliorated dyslipidemia, hepatic steatosis, and insulin resistance in HFD-fed mice (15). Api was also effective in alleviating obesity-related inflammation (14–16). For instance, Api treatment reduced inflammatory markers and immune cell infiltration in the liver and adipose tissue of obese mice (14, 16). Furthermore, Api has been shown to reduce systemic levels of pro-inflammatory cytokines in obese mice (15, 16). By acting as a modulator of PPARγ, Api reduces NF-κB activation and promotes M2 macrophage polarization (16). Moreover, our group has previously shown that Api attenuates IL-1β-induced inflammation and promotes beige adipocyte development in primary human adipocyte cultures through mechanisms linked to PGE2-EP4 signaling (17).

The present study was designed to evaluate the *in vivo* effects of Api on activating adaptive thermogenesis in the presence of inflammation. We evaluated changes in adipocyte browning and lipid dynamics following Api supplementation in peripheral adipocytes. Additionally, we examined potential alterations in skeletal adipocytes and bone parameters following Api supplementation. Our results indicate that Api is effective in promoting iWAT browning and reducing inflammation in LPS-injected mice. Additionally, Api altered lipid turnover in peripheral fat and induced bone marrow adipose tissue (BMAT) expansion without negatively impacting bone parameters.

## 2 Materials and methods

### 2.1 Reagents and chemicals

The β3-adrenergic receptor (β3-AR) agonist CL 316,243, Dimethyl Sulfoxide (DMSO), and LPS from *Escherichia coli* O111:B4 (3×10^6^ EU [endotoxin units]/mg) were purchased from Sigma-Aldrich. Api was purchased from Selleck Chemicals and dissolved in DMSO. TRIzol reagent and DNase treatment kits were purchased from Invitrogen. Fast SYBR green Master Mix and the cDNA synthesis kit were obtained from Applied Biosystems.

### 2.2 Animals

All experimental protocols and procedures were approved by the Institutional Research Ethics Committee (REC) of King Saud University, Riyadh, Saudi Arabia (Approval No. KSU-SE-18-39). C57BL/6 male mice from Taconic Biosciences, USA, were housed at a room temperature of 22-24 °C with 45% humidity under a 12 h light/dark cycles. At 6–8 weeks of age, mice (n = 8–11 per group) were injected intraperitoneally with 100 µl of either vehicle (0.9% NaCl+ DMSO), a low dose of *Escherichia coli* LPS (8 μg/mouse, equivalent to 24,000 EU), or LPS + Api (30 mg/kg body weight) every other day for 2 weeks. The dose and duration of LPS were selected based on our previously published study (4) and relevant prior literature (18). The dose and duration of Api treatment were selected based on previous studies (14, 16) demonstrating significant anti-inflammatory effects at this level. To stimulate adaptive thermogenesis, mice received intraperitoneal (i.p.) injections of CL316,243 (CL) at a dose of 1 mg/kg body weight for the last 5 days before necropsy. Anesthesia was induced by inhalation of a 30% v/v isoflurane solution in propylene glycol using the open-drop method, and the animals were subsequently euthanized by cervical dislocation. At necropsy, adipose tissues were collected, snap-frozen in liquid nitrogen, and stored at − 80 °C until analysis.

### 2.3 Body temperature measurements

Core body temperature was measured using a rectal probe inserted into the anal cavity of adult mice and recorded with a digital monitor (CODA, Kent Scientific Corp). The average of three readings per mouse was calculated. For surface body temperature, a digital infrared thermometer (BT-985C, BTMETER) was used, and the average of three readings per mouse was calculated.

### 2.4 Blood sample analysis

Plasma levels of non-fasting triglycerides (TG) and free fatty acids (FFA) were measured using enzymatic colorimetric assay kits (Sigma-Aldrich), according to the manufacturer’s instructions. Glucose levels were measured using a commercial ELISA kit (MyBioSource), following the manufacturer’s instructions, with plasma samples obtained from non-fasting animals.

### 2.5 Quantitative real-time polymerase chain reaction

Total RNA was extracted using TRIzol reagent and isolated using the chloroform-isopropanol method. To remove genomic DNA contamination, RNA was purified with DNase treatment (Invitrogen). Reverse transcription was performed using 2 µg of RNA with the High-Capacity cDNA Reverse Transcription Kit. Gene expression was assessed by real-time qPCR (ViiA 7, Applied Biosystems). Data were normalized to 36B4 or Gapdh, and relative gene expression levels were calculated. Gene-specific primers for qPCR were obtained from Macrogen (Seoul, South Korea), and the primer sequences are listed in **Supplementary Table S1**.

### 2.6 Hematoxylin & Eosin staining

Adipose tissue samples from C57BL/6 mice were immediately fixed in 10% buffered formalin. Paraffin-embedded adipose tissues were sectioned into 10 μm-thick slices for H&E staining. Tibiae were cleaned and fixed in 10% buffered formalin for 48–72 hours, then decalcified in 14% EDTA (pH 7.4) before paraffin embedding and H&E staining.

### 2.7 Mitochondrial DNA quantification by qPCR

Genomic DNA was extracted from mouse subcutaneous adipose tissue using DNAzol (Life Technologies). Quantitative PCR was performed in duplicate using mitochondrial DNA (mtDNA)-specific primers (16S rRNA) and nuclear DNA-specific primers (hexokinase 2), as described previously (4, 19). Results were calculated based on the difference in threshold cycle values between mtDNA and nuclear DNA amplification.

### 2.8 Western blot analysis

Total protein lysates from adipose tissues were obtained using ice-cold RIPA lysis buffer (Thermo Fisher Scientific) supplemented with protease and phosphatase inhibitors. Proteins were separated using 10% SDS-PAGE gels, transferred to PVDF membranes using either the Trans-Blot Turbo Transfer System (Bio-Rad) or wet electroblotting (Bio-Rad), and incubated with the appropriate primary antibodies. Antibodies against p-HSL (Ser660) and UCP1 were obtained from Thermo Fisher Scientific, and the GAPDH antibody was obtained from Cell Signaling Technology. Blots were visualized using the ChemiDoc MP Imaging System (Bio-Rad).

### 2.9 Micro-computed tomography scanning

Tibiae were collected, and all soft tissues were removed. The bones were then fixed in 10% buffered formalin for 3 days and scanned using an X-ray micro-CT system (Skyscan 1172, Bruker) at the Engr. Abdullah Bugshan Research Chair for Dental and Oral Rehabilitation, King Saud University.

### 2.10 Statistical analysis

For comparisons between two groups, data were analyzed using Student’s t test. For comparisons involving more than two groups, one-way ANOVA followed by Tukey’s multiple comparison test was used. All data are presented as mean ± SEM. *P<0.05, **P<0.01, ***P <0.001, ****P<0.0001 were considered statistically significant, as determined by Student’s t-test. Values not sharing a common letter differ significantly (P<0.05), as determined by one-way ANOVA. All statistical analyses were conducted using GraphPad Prism 7 (Version 7.03).

## 3 Results

### 3.1 Apigenin restores CL-induced adaptive thermogenesis in LPS-injected mice

**Figure 1:**
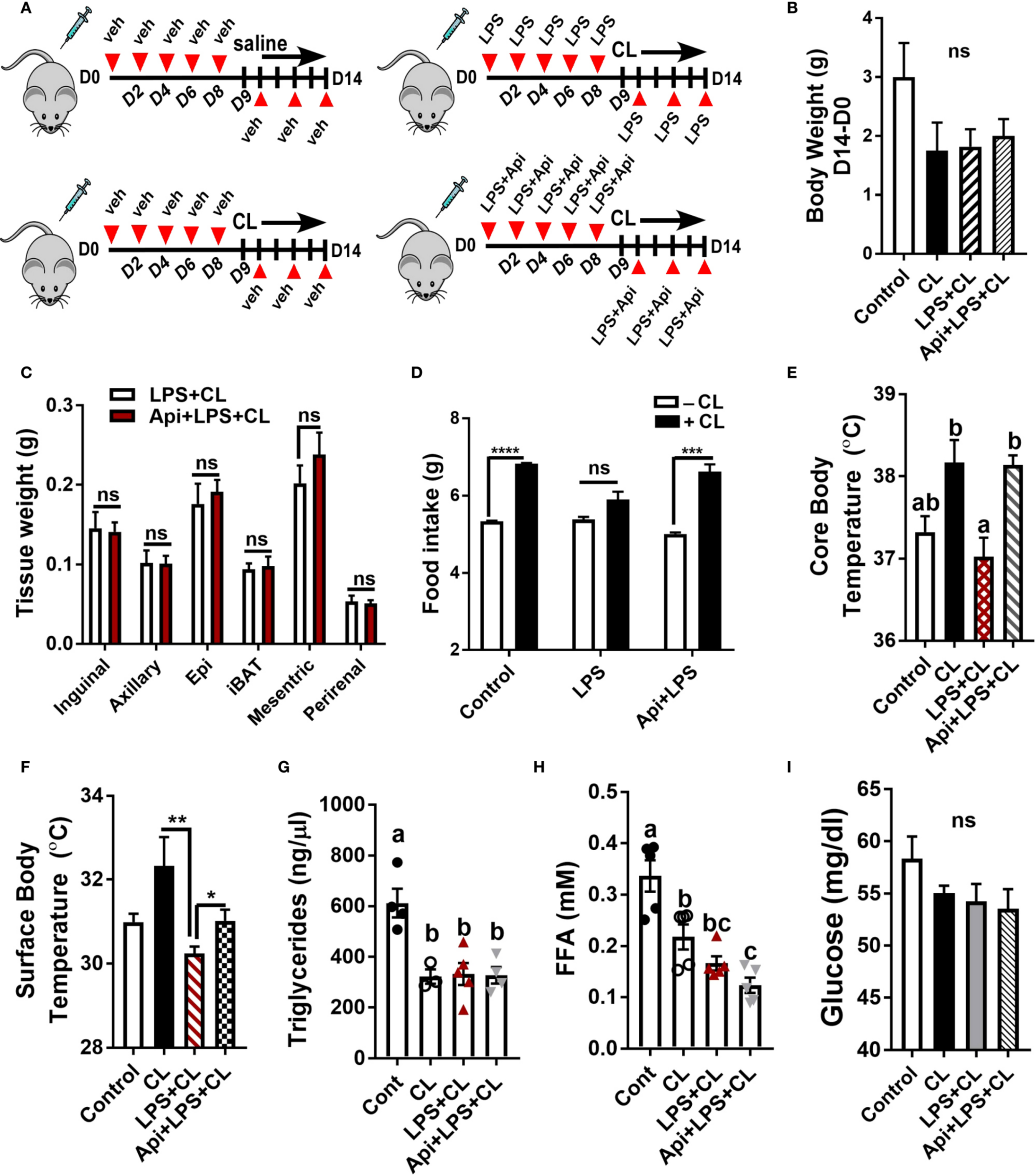
Apigenin restores CL-induced adaptive thermogenesis in LPS-injected mice. C57BL/6 mice (n = 8-11) received i.p. injections of either vehicle, LPS, or LPS + Api every other day for 2 weeks. For the last 5 days, animals received i.p. injections of CL, while control mice received saline. **(A)** Schematic representation of the animal experiment. **(B)** Changes in body weight. **(C)** Adipose tissue wet weight. **(D)** Average daily food intake measured before and after CL treatment. **(E)** Core body temperature measured by rectal thermometer at the end of the experiment. **(F)** Surface body temperature measured by infrared thermometer at the end of the experiment. **(G-I)** Non-fasting plasma triglyceride **(G)**, free fatty acid **(H)**, and glucose **(I)** levels. All data are presented as mean ± SEM. *P<0.05, **P<0.01, ***P<0.001, ****P<0.0001 by Student’s t-test. Values not sharing a common letter differ significantly (P<0.05) by one-way ANOVA. ns, not significant.

To investigate the protective effect of Api on adaptive thermogenesis under inflammatory conditions, C57BL/6 mice were injected with either vehicle, LPS, or LPS + Api over a period of 2 weeks. During the final 5 days, all mice except those in the control group were treated with CL to activate adaptive thermogenesis (**Figure 1A**). CL treatment induced a modest weight reduction in all treated groups compared to the control group (without CL), with no significant differences among the CL-treated groups (**Figure 1B**). Api administration did not significantly affect adipose tissue weight in the presence of LPS+CL (**Figure 1C**), but it restored the CL-induced increase in food intake, which was suppressed in the LPS group (**Figure 1D**). The lack of changes in body weight and fat tissue weight in Api-supplemented mice, despite increased food intake, likely indicates activation of adaptive thermogenesis. To investigate this, we measured core and surface body temperatures and found that Api supplementation in LPS+CL-treated mice significantly increased both, compared to the LPS+CL group (**Figures 1E, F**). Although CL treatment led to a significant decrease in plasma TG and FFA levels, as well as a modest reduction in glucose levels across all CL-treated groups compared to the control group (**Figures 1G–I**), Api supplementation did not significantly affect these parameters compared to the LPS+CL group. These data suggest that Api supplementation restores CL-induced adaptive thermogenesis in the presence of LPS.

### 3.2 Apigenin attenuates inflammation and enhances CL-induced iWAT browning and lipid metabolism in LPS-injected mice

**Figure 2:**
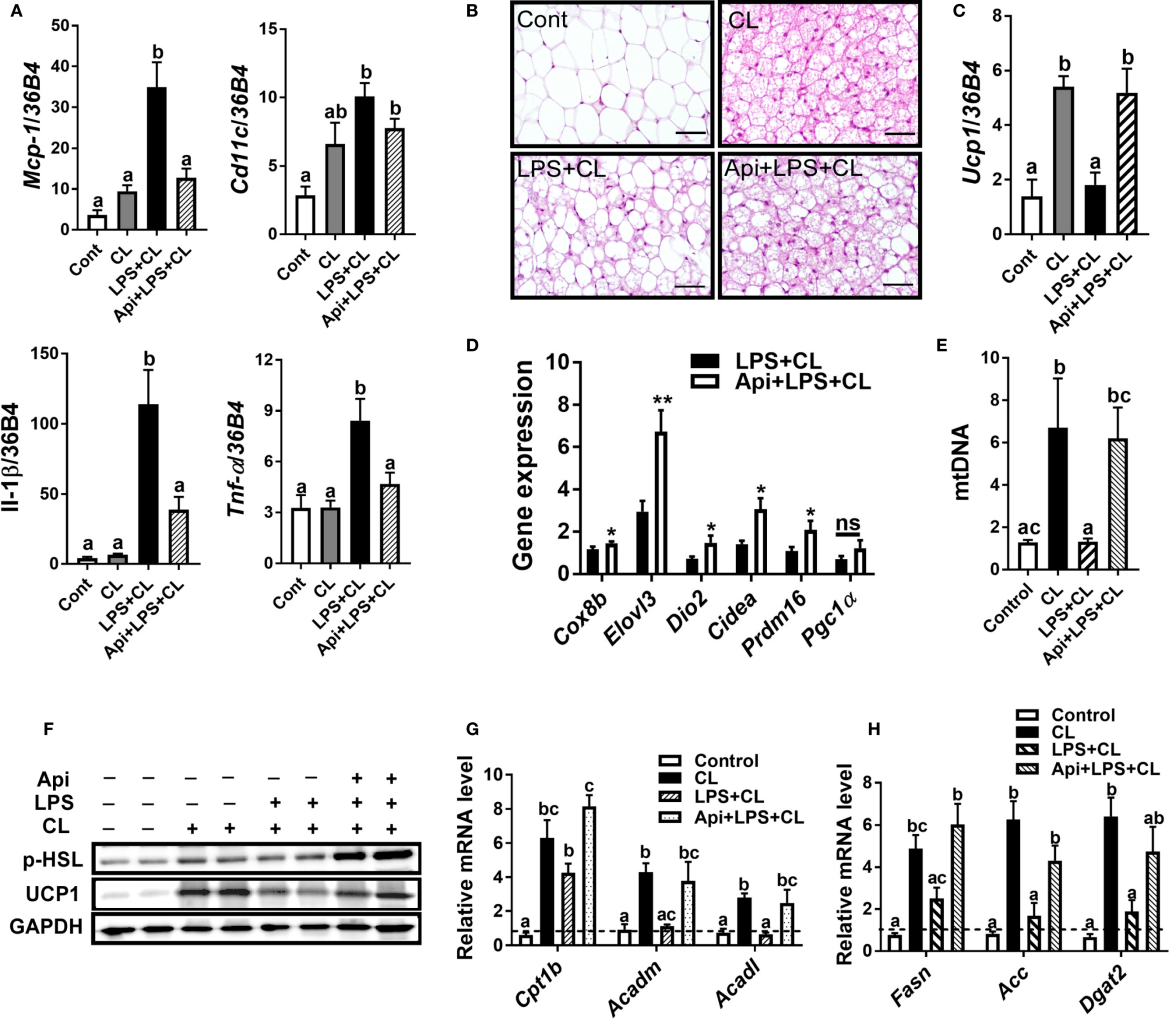
Apigenin attenuates inflammation and promotes CL-induced iWAT browning in LPS-injected mice. **(A)** Relative gene expression analysis of inflammatory markers in inguinal fat. **(B)** Representative images of H&E staining from inguinal fat (scale bar = 50 μm). **(C, D)** Relative gene expression analysis of UCP1 **(C)** and other thermogenic markers **(D)** in iWAT. **(E)** Relative mtDNA content in subcutaneous fat. **(F)** Western blot analysis of inguinal fat; full-length blots are presented in **Supplementary Figure S3**. **(G, H)** Relative gene expression analysis of genes related to fatty acid oxidation **(G)** and lipogenesis **(H)**. All data are presented as mean ± SEM. *P<0.05, **P<0.01 by Student’s t-test. Values not sharing a common letter differ significantly (P<0.05) by one-way ANOVA. Mcp-1; monocyte chemotactic protein-1; Ucp1, uncoupling protein 1; Dio2, iodothyronine deiodinase 2; Cidea, cell death-inducing DFFA-like effector A; and Pgc1α, peroxisome proliferator-activated receptor gamma coactivator 1-alpha. ns, not significant.

We examined iWAT to determine whether Api supplementation restores CL-induced adaptive thermogenesis in LPS-injected mice by suppressing inflammation and promoting iWAT browning. The gene expression of inflammatory markers Mcp-1, Il-1β, and Tnf-α in iWAT was significantly reduced by Api administration in LPS-injected animals (**Figure 2A**). Api treatment was also associated with an increase in brown-like morphology (**Figure 2B**). Additionally, mice receiving Api+LPS+CL showed a significant upregulation in the gene expression of Ucp1 (**Figure 2C**) and other brown fat-specific markers, including Cox8b, Elovl3, Dio2, Cidea, and Prdm16 (**Figure 2D**), compared to the LPS+CL group. Furthermore, the CL-induced increase in mtDNA content, which was suppressed by LPS, was restored by Api treatment (**Figure 2E**). Protein levels of p-HSL and UCP1 were also elevated following Api administration in LPS-injected mice (**Figure 2F**). Moreover, compared to the LPS+CL group, mice treated with Api+LPS+CL exhibited higher expression of genes related to fatty acid oxidation (**Figure 2G**) and lipogenesis (**Figure 2H**). These data indicate that Api reduces inflammation, promotes iWAT browning, and enhances lipid metabolism in the iWAT of mice treated with LPS and CL.

### 3.3 The effects of apigenin on thermogenesis and lipid metabolism detected in iWAT were not observed in iBAT or eWAT

**Figure 3:**
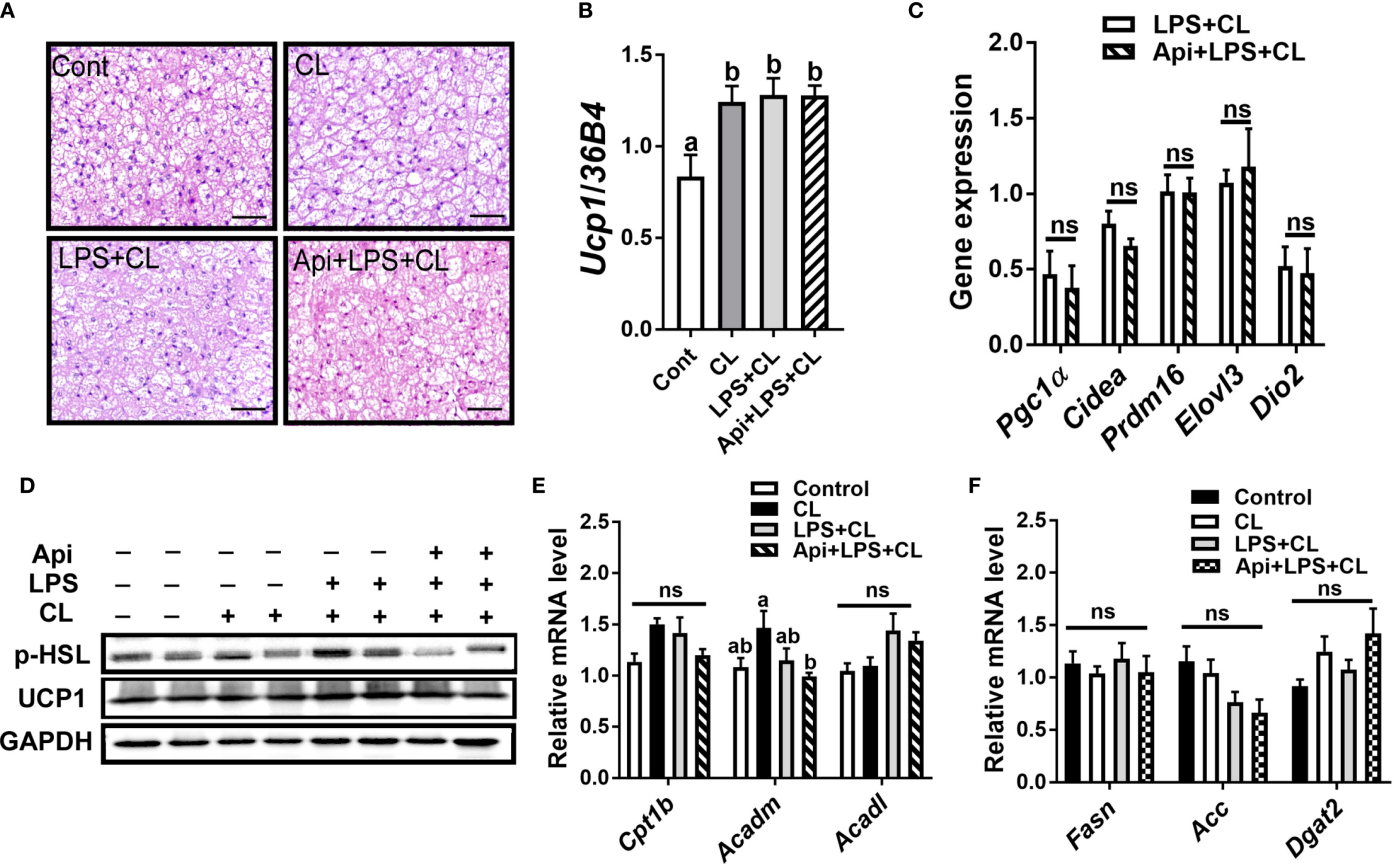
Apigenin has no effect on thermogenesis or lipid metabolism in the iBAT of LPS-injected mice during CL stimulation. **(A)** Representative images of H&E staining from iBAT (scale bar = 50 μm). **(B, C)** Relative gene expression analysis of UCP1 **(B)** and other thermogenic markers **(C)** in iBAT. **(D)** Western blot analysis of iBAT; full-length blots are presented in **Supplementary Figure S4**. **(E, F)** Relative gene expression analysis of genes related to fatty acid oxidation **(E)** and lipogenesis **(F)**. All data are presented as mean ± SEM. Values not sharing a common letter differ significantly (P<0.05) by one-way ANOVA. ns, not significant.

**Figure 4:**
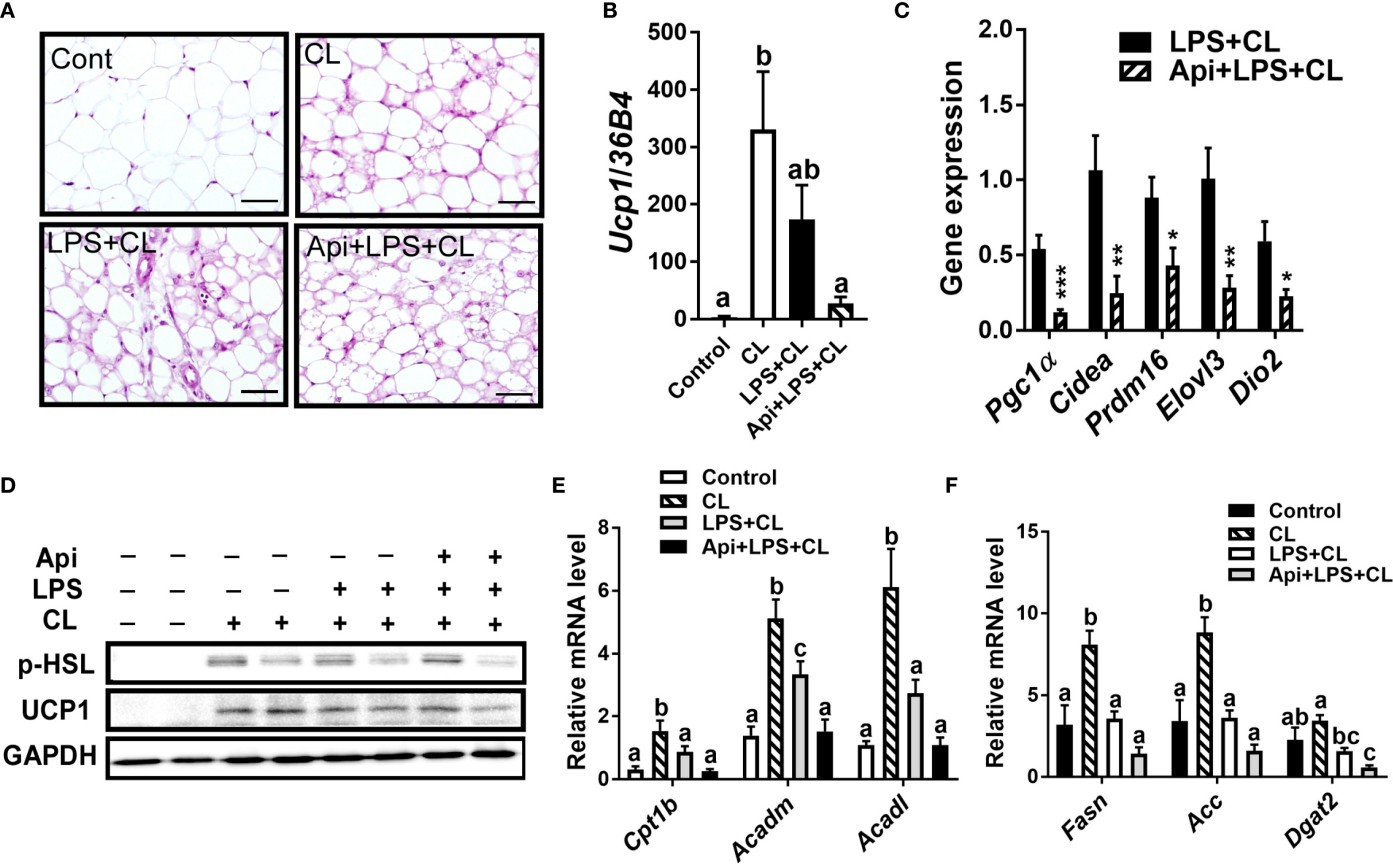
Apigenin has no positive impact on thermogenesis or lipid metabolism in the eWAT of LPS-injected mice during CL stimulation **(A)** Representative images of H&E staining from eWAT (scale bar = 50 μm). **(B, C)** Relative gene expression analysis of UCP1 **(B)** and other thermogenic markers **(C)** in eWAT. **(D)** Western blot analysis of eWAT; full-length blots are presented in **Supplementary Figure S5**. **(E, F)** Relative gene expression analysis of genes related to fatty acid oxidation **(E)** and lipogenesis **(F)**. All data are presented as mean ± SEM. *P<0.05, **P<0.01, ***P<0.001 by Student’s t-test. Values not sharing a common letter differ significantly (P<0.05) by one-way ANOVA.

We evaluated whether the thermogenic effects of Api observed in iWAT are reproducible in other fat depots. Administration of Api had no effect on adipocyte morphology in interscapular brown adipose tissue (iBAT) of LPS+CL-treated mice (**Figure 3A**). Additionally, Api did not affect the gene expression levels of Ucp1 (**Figure 3B**) or other thermogenic markers (**Figure 3C**) in iBAT. Furthermore, Api treatment did not increase the protein expression levels of p-HSL and UCP1 in iBAT (**Figure 3D**). Moreover, no changes were observed in the expression of genes related to fatty acid oxidation or *de novo* lipid synthesis upon Api supplementation (**Figures 3E, F**). In epididymal white adipose tissue (eWAT), administration of Api with LPS did not induce brown-like morphology compared to LPS treatment in CL-injected mice (**Figure 4A**). In fact, Api negatively regulated the gene expression levels of UCP1 (**Figure 4B**) and other thermogenic markers (**Figure 4C**) in LPS+CL-treated mice. No changes were observed in the protein levels of p-HSL or UCP1 in eWAT following Api+LPS treatment compared to the LPS group in CL-injected mice (**Figure 4D**). Additionally, Api negatively regulated the gene expression of key enzymes involved in fatty acid oxidation (**Figure 4E**) and *de novo* lipid synthesis (**Figure 4F**) in eWAT of LPS + CL-injected mice compared to the LPS + CL group; however, these decreases were not statistically significant for all genes tested. These data indicate that the browning effect of Api during CL stimulation is specific to iWAT in LPS-injected mice. Therefore, the enhanced adaptive thermogenesis observed in the Api + LPS group of CL-stimulated mice was primarily mediated by iWAT browning.

### 3.4 Apigenin alone does not induce thermogenesis in the absence of LPS and CL stimulation

**Figure 5:**
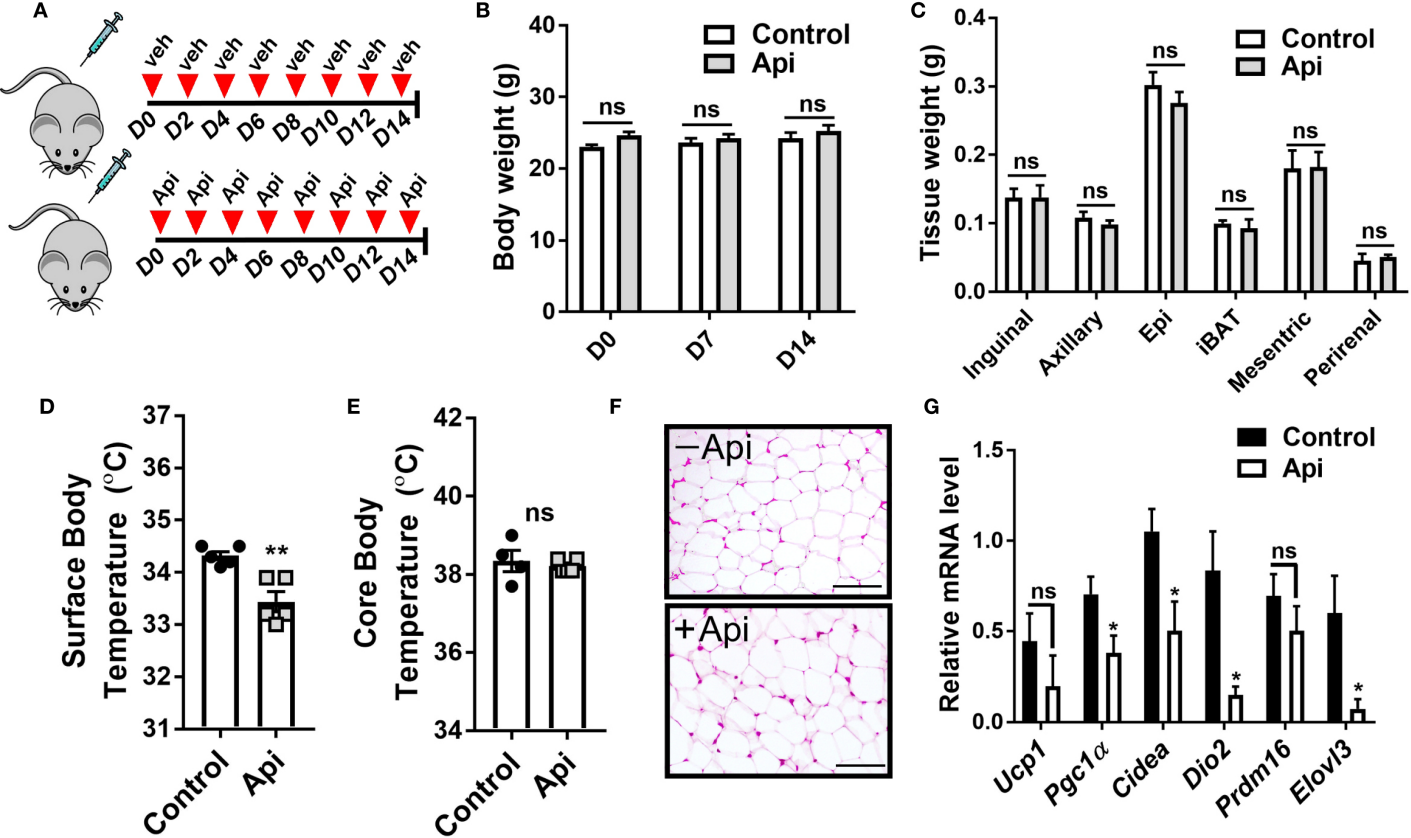
Apigenin imposes no thermogenic effects in the absence of CL and LPS. C57BL/6 mice (n = 5) received i.p. injections of either vehicle or Api every other day for 2 weeks. **(A)** Schematic representation of the injection schedule. **(B)** Body weight on day 0, 7, and 14. **(C)** Adipose tissue wet weight. **(D)** Surface body temperature measured by infrared thermometer at the end of the experiment. **(E)** Core body temperature measured by rectal thermometer at the end of the experiment. **(F)** Representative images of H&E staining from iWAT (scale bar = 25 μm). **(G)** Relative gene expression analysis of thermogenic markers in iWAT. All data are presented as mean ± SEM. *P<0.05, **P<0.01 by Student’s t-test. ns, not significant.

While Api was capable of reversing the negative effects of LPS on iWAT browning in CL-injected mice, it remained unclear whether Api alone could induce iWAT browning without any thermogenic stimuli. To address this, C57BL/6 mice were injected with either DMSO or Api for 2 weeks (**Figure 5A**). No differences in body weight (**Figure 5B**) or fat tissue weight (**Figure 5C**) were observed between the two groups. Additionally, Api treatment did not increase surface (**Figure 5D**) or core body temperature (**Figure 5E**). Histological analysis showed no brown-like morphology in iWAT of Api-treated mice (**Figure 5F**). Furthermore, Api did not upregulate thermogenic markers in iWAT (**Figure 5G**). Overall, since Api alone was not capable of stimulating iWAT browning, it likely improves CL-induced iWAT browning in LPS-injected mice by interfering with LPS-associated mechanisms responsible for inhibiting iWAT browning.

### 3.5 Apigenin promotes BMAT accumulation during adaptive thermogenesis in LPS-injected mice without affecting bone parameters

**Figure 6:**
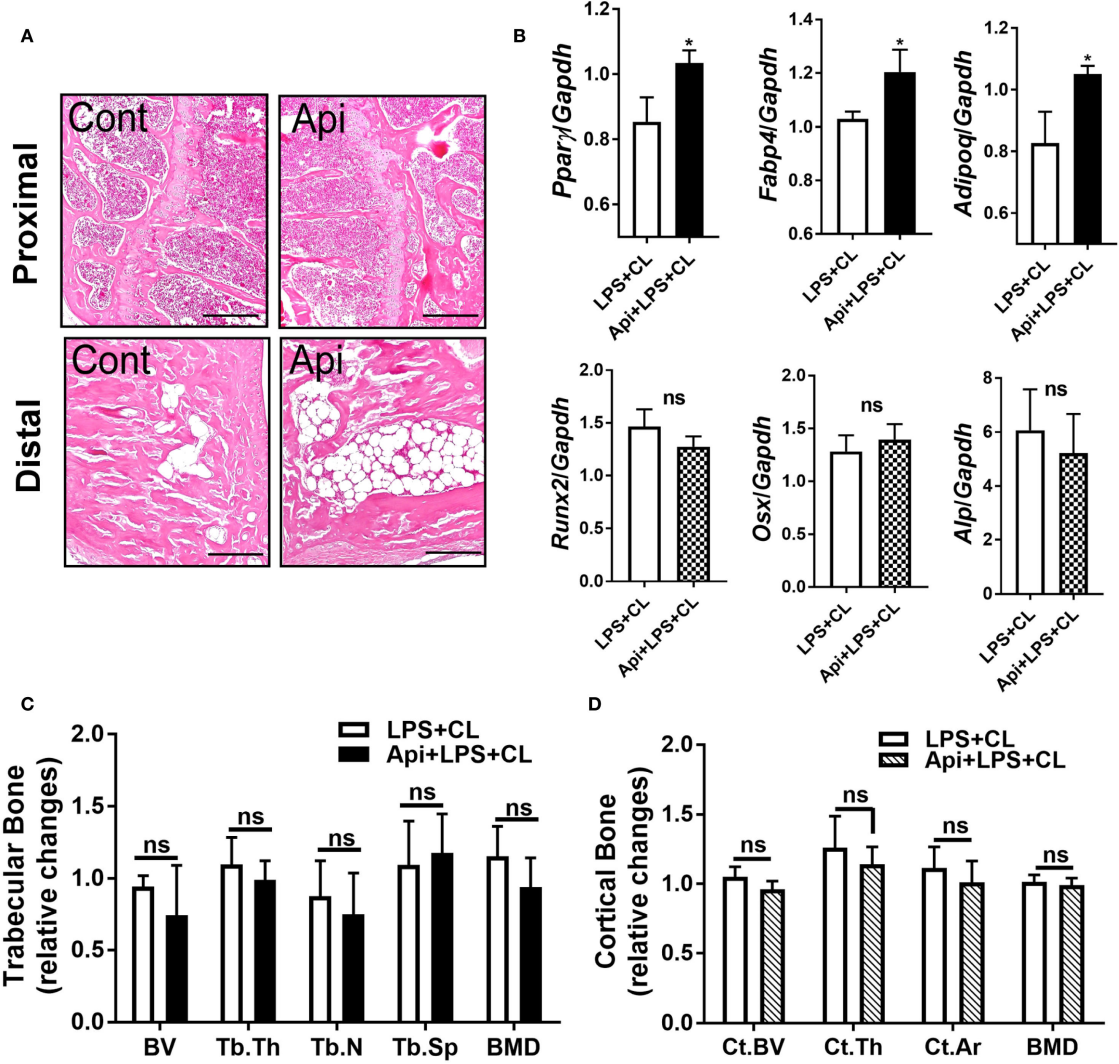
Apigenin induces BMAT formation in LPS-injected mice during adaptive thermogenesis without causing negative effects on bone parameters. C57BL/6 mice received either LPS or LPS + Api every other day for 2 weeks. For the last 5 days, all animals were subjected to β3-AR activation using CL. **(A)** Representative images of H&E staining from tibiae (scale bar = 20 μm). **(B)** Relative gene expression analysis of adipogenic markers (top panel) in BM and osteogenic markers (bottom panel) in the tibiae (n = 4-6). **(C, D)** µCT analysis of proximal trabecular **(C)** and cortical **(D)** tibial bone (n=6). All data are presented as mean ± SEM. *P<0.05 by Student’s t-test. BV, bone volume; Tb.Th, trabecular thickness; Tb.N, trabecular number; Tb.Sp, trabecular separation; BMD, bone mineral density; Ct.BV, cortical bone volume; Ct.Th, cortical thickness; and Ct.Ar, cortical area. ns, not significant.

**Figure 7:**
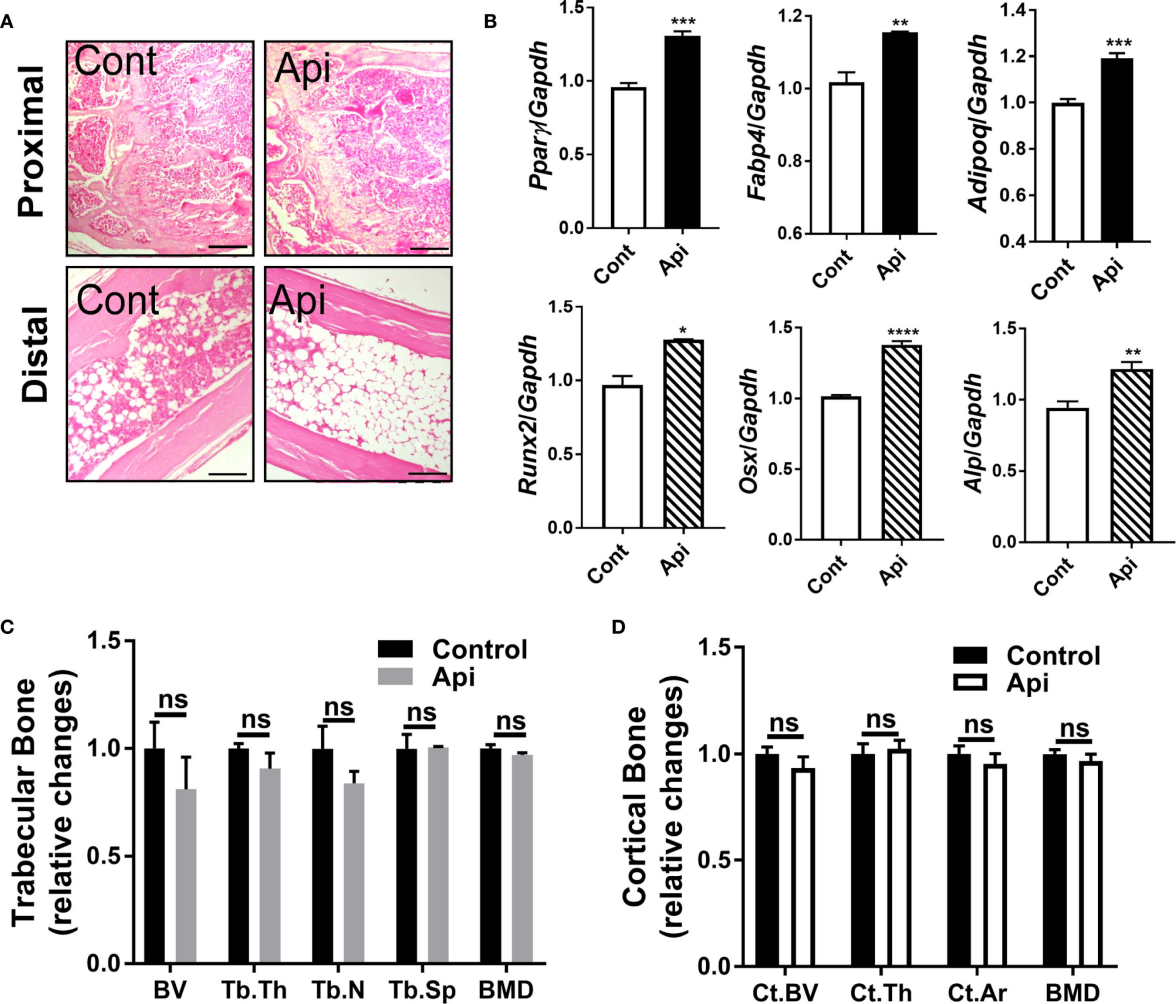
Apigenin alone stimulates both adipogenic and osteogenic markers in mouse tibiae without altering bone parameters. C57BL/6 mice were given either a vehicle or Api every other day for 2 weeks. **(A)** Representative images of H&E staining for tibiae (scale bar = 20 μm). **(B)** Relative gene expression analysis of adipogenic markers (top panel) in BM and osteogenic markers (bottom panel) in the tibiae (n = 5). **(C, D)** µCT analysis of proximal trabecular **(C)** and cortical **(D)** tibial bone (n = 5). All data are presented as mean ± SEM. *P<0.05, **P<0.01, ***P<0.001, ****P,0.0001 by Student’s t-test. ns, not significant.

Given that Api has varying effects on peripheral adipose tissue, we investigated whether it also affects BMAT by examining the tibiae of C57BL/6 mice treated with either LPS alone or Api + LPS, followed by CL treatment. Histological analysis of the tibiae revealed a robust increase in BMAT content following Api treatment (**Figure 5A**). RNA was isolated from the bone marrow (BM) and tibiae of the mice. Analysis of BM showed a significant increase in adipogenic gene expression (Pparγ, Fabp4, and Adipoq) in the Api + LPS group compared to the LPS group (**Figure 6**, top panel). In contrast, the expression of osteogenic markers in the tibiae (Runx2, Osx, and Alp) was not different between the Api +LPS and LPS groups (**Figure 6**, bottom panel). Despite the increase in BMAT content and adipogenic markers in the Api + LPS group, micro-CT analysis indicated no significant effects on the microarchitecture of either trabecular (**Figure 6C**) or cortical bone parameters (**Figure 6D**). The observed increase in BMAT and adipogenic markers in the bone microenvironment of Api + LPS-injected mice undergoing adaptive thermogenesis appears to be independent of iWAT browning. This is supported by the fact that animals treated with Api alone, which did not show increased thermogenesis, also exhibited higher BMAT content (**Figure 7A**) and a significant increase in adipogenic markers in their BM (**Figure 7B**, top panel) compared to control counterparts. Surprisingly, Api treatment alone had a positive effect on the expression of osteogenic marker genes (**Figure 7B**, bottom panel). However, micro-CT analysis revealed no changes in either trabecular (**Figure 7C**) or cortical bone parameters (**Figure 7D**) in the Api-only group.

To better understand the broader effects of β3-AR activation and inflammation on bone homeostasis, we next examined changes in gene expression and bone structure in these experimental conditions. Our findings show that β3-AR activation in mice suppressed adipogenic markers (**Supplementary Figure S1A**) in the BM and modestly induced osteogenic markers (**Supplementary Figure S1B**) in the tibiae, without affecting bone parameters (**Supplementary Figures S1C, D**). By contrast, the presence of LPS in animals undergoing CL stimulation caused no changes in the expression of adipogenic (**Supplementary Figure S2A**) or osteogenic markers (**Supplementary Figure S2B**) in bone, but resulted in a significant decrease in BV, Tb.N, and BMD of the trabecular bone (**Supplementary Figure S2C**), without affecting cortical bone parameters (**Supplementary Figure S2D**). Together, these data suggest that Api treatment reverses CL-induced downregulation of adipogenic markers in the BM and attenuates LPS-induced deterioration of trabecular bone parameters.

## 4 Discussion

**Figure 8:**
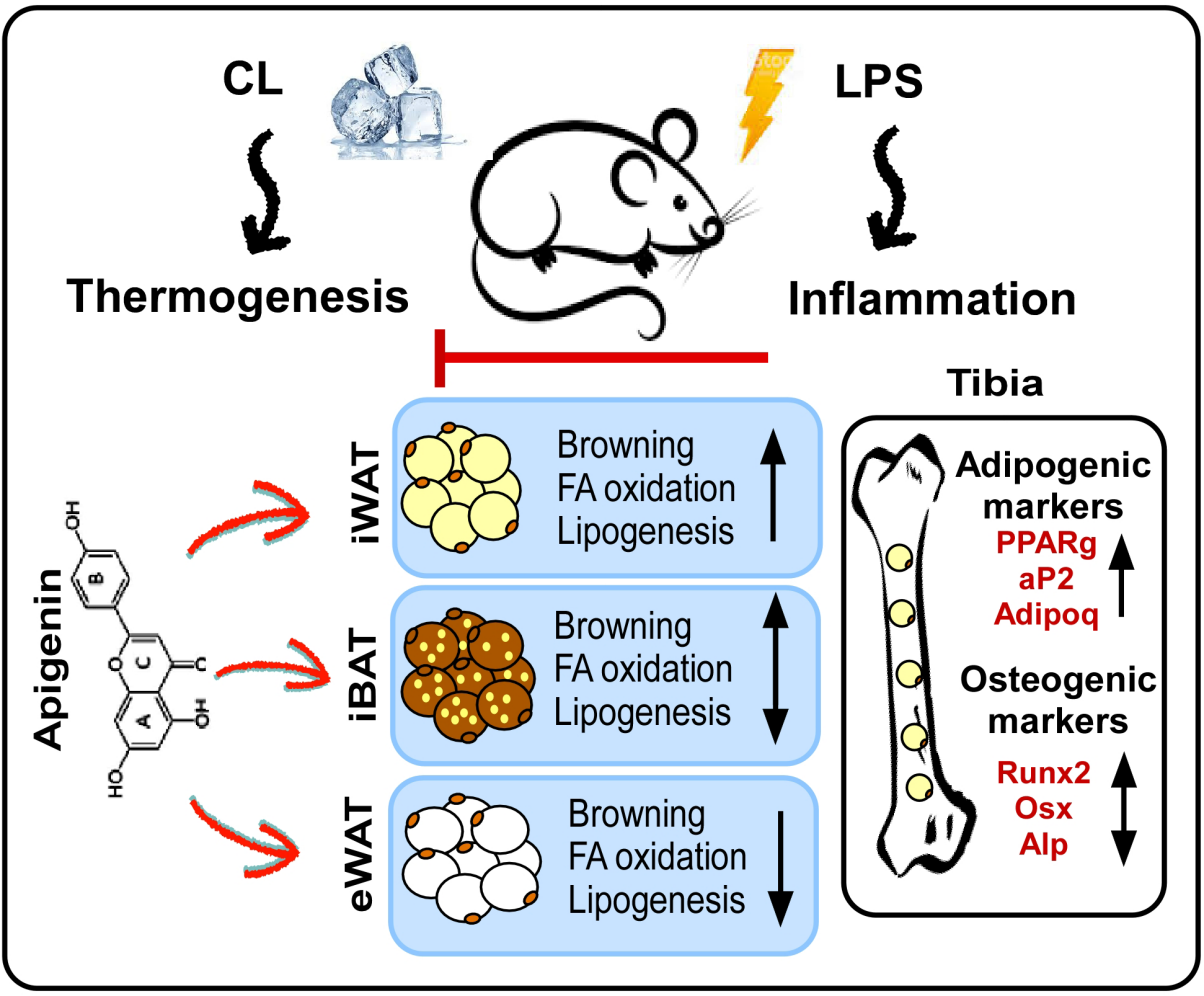
Proposed model for the effects of apigenin on peripheral and bone marrow fat in LPS-injected mice undergoing adaptive thermogenesis. LPS attenuates CL-induced thermogenesis, while Api administration restores CL-induced thermogenesis in LPS-injected mice by reducing inflammation and promoting the browning of iWAT. Api-induced iWAT browning is associated with increased expression of genes related to fatty acid oxidation and lipogenesis. The thermogenic and lipid turnover effects of Api were not observed in iBAT and were suppressed in eWAT. Api-induced changes in peripheral fat were accompanied by increased formation of BMAT, without significantly impacting trabecular or cortical bone parameters.

The present study elucidates the potential of Api in rescuing adaptive thermogenesis under inflammatory conditions in mice. As illustrated in our proposed model (**Figure 8**), Api attenuates inflammation and enhances adaptive thermogenesis by promoting iWAT browning. Api-induced iWAT browning was accompanied by increased expression of genes involved in fatty acid oxidation and *de novo* fatty acid synthesis. Interestingly, no changes in thermogenesis or lipid metabolism were observed in iBAT, while these processes were reduced in eWAT. Thus, the restoration of thermogenesis by Api under inflammatory conditions appears to be primarily mediated through its effects on iWAT. Additionally, Api promoted BMAT expansion without altering bone parameters in LPS-injected mice undergoing adaptive thermogenesis.

The thermogenic effects of Api on adipocytes have been reported by our group and others (17, 20–22). In a previous study, we demonstrated that Api treatment of primary human adipocytes restores dibutyryl-cAMP-induced browning and enhances mitochondrial content and function, regardless of the presence of IL-1β. This browning effect of Api was mediated through mechanisms involving the repression of NF-κB activation, as well as the activation of COX2/PGE2 axis and EP4 signaling (17). Additionally, a study reported that cross-talk between endothelial cells and adipocytes mediates Api-induced adipocyte browning through mechanisms involving VEGF stimulation (20). A recent study by Xiong et al. also demonstrated that Api promotes the browning of white adipocytes in obese mice, as well as in 3T3-L1 and primary mouse adipocytes. This adipocyte browning effect was attributed to the inhibitory effect of Api on autophagy, mediated by activation of the PI3K-Akt-mTOR pathway (21). Furthermore, the use of Api encapsulated within nanoparticles was recently shown to induce immunomodulation and promote the expression of thermogenic markers in the adipose tissue of obese mice (22).

In the current study, our data demonstrate that Api is effective in restoring CL-induced adaptive thermogenesis in LPS-injected mice, as evidenced by increased core and surface body temperatures (**Figures 1E, F**). Moreover, our results revealed enhancements in brown-like morphology, brown-specific gene expression, mtDNA content, and expression levels of UCP1 and p-HSL proteins (**Figures 2B-F**). Consistent with our findings on the thermogenic potency of Api, Xiong et al. reported that Api supplementation in HFD-fed mice effectively enhanced the expression of brown-specific markers compared to HFD controls and increased core body temperature upon cold exposure (21). Although Xiong et al. reported a substantial reduction in body weight and adipose tissue mass following Api administration in obese mice, our results showed no effect of Api supplementation on either body weight or adipose tissue mass. This lack of difference may be attributed to the use of a normal chow diet in our study and the fact that CL injections effectively reduced body weight across all groups (**Figure 1B**). However, the Api+LPS group exhibited increased food intake without a corresponding gain in body weight after CL stimulation, compared to the LPS group, indicating thermogenic activation and energy dissipation upon Api supplementation (**Figure 1D**). The inclusion of indirect calorimetry in future studies would enable a more comprehensive assessment of whole-body energy metabolism in response to Api supplementation.

Previous reports have shown that Api affects lipid metabolism and reduces lipid accumulation in the liver and adipose tissue (14, 15, 21). In adipocyte cultures, TG accumulation was decreased upon Api treatment in a dose-dependent manner (21). In obese mice, circulating levels of FFA, TG, total cholesterol, and apolipoprotein B were reduced following Api treatment (15, 21). In our LPS+CL-injected mice, Api supplementation increased lipid turnover, as reflected by the induced expression of genes related to both fatty acid oxidation and *de novo* synthesis (**Figures 2G, H**). The activation of lipid turnover observed in our mice may be linked to the browning effects of Api, as lipids are essential substrates for thermogenesis (23). In support of this, impaired thermogenesis in ob/ob mice has been associated with reduced expression of genes involved in fatty acid synthesis, mobilization, and oxidation, leading to diminished lipid utilization and limited substrate availability for thermogenesis (23).

The beneficial effects of Api on thermogenesis in LPS-injected mice were not evident in other fat depots. We observed no changes in brown-like morphology, thermogenic markers, or lipid dynamics in iBAT following Api supplementation in LPS-injected mice (**Figures 3A-F**), possibly because BAT is more resistant to inflammation than other fat depots, except under conditions of severe obesity (23). In eWAT, however, brown-like morphology, thermogenic markers, and lipid turnover were all suppressed by Api in LPS-injected mice (**Figures 4A-F**). The depot-specific responses to Api are consistent with findings from primary adipocytes isolated from iWAT and eWAT in a previous study, which may be explained by the lower susceptibility of eWAT to browning compared to iWAT (21). The diminished browning response in eWAT may be attributed to its lower sympathetic innervation compared to subcutaneous fat (24, 25). Furthermore, visceral fat exhibits a more pronounced pro-inflammatory profile (26) and reduced angiogenic capacity (27) compared to subcutaneous depots. These differences may contribute to the reduced responsiveness of eWAT to Api-induced thermogenesis and lipid turnover under inflammatory conditions.

BMAT is a distinctive fat depot located near bone surfaces. BMAT responds to changes in whole-body energy metabolism and plays diverse regulatory roles in skeletal biology (28, 29). In the current study, changes in BMAT following Api supplementation were examined. The results showed that Api supplementation induced BMAT accumulation in LPS-injected mice undergoing thermogenic stimulation by CL (**Figures 6A, B**, top). Although BMAT was induced in these mice, there were no changes in the expression of osteogenic markers in the tibiae (**Figure 6B**, bottom) and no significant alterations in bone microarchitecture parameters (**Figures 6C, D**). The precise role of this BMAT increase in relation to the effects of Api on peripheral fat depots remains to be elucidated. However, our findings indicate that the effect of Api on BMAT accumulation appears to be independent of its effects on iWAT browning in LPS-injected mice, as Api treatment alone, in the absence of LPS and CL, also increased BMAT accumulation (**Figure 7A**).

Flavonoids can modulate self-renewal and osteogenic differentiation potential of mesenchymal stem cells through several mechanisms. These include activation of signaling pathways such as Wnt/β-catenin, ERK, and PI3K/Akt, as well as regulation of bone-specific markers and transcription factors. Additionally, their well-known antioxidant and anti-inflammatory properties may further contribute to their osteogenic effects [reviewed in (30)]. Multiple lines of evidence suggest that Api has positive effects on bone metabolism. In OVX mice, Api decreased trabecular bone loss without altering cortical bone loss (31). In obese mice, the supplementation with Api for 21 days increased the trabecular bone volume ratio (16). Furthermore, Api has been identified as an enhancer of osteoblastic differentiation in human bone marrow stromal stem cells. This osteogenic effect was observed even in cells derived from elderly female participants and during ex vivo treatment of organotypic embryonic check femur cultures (32). Consistent with these findings, the current study demonstrated that Api treatment alone induced both adipogenic and osteogenic markers in the bone microenvironment (**Figure 7B**), indicating a dual regulatory effect on bone marrow cell differentiation. However, despite the upregulation of osteogenic gene expression in these mice, no significant changes were observed in trabecular or cortical bone parameters (**Figures 7C, D**), possibly due to the relatively short duration of Api supplementation, which may have been insufficient to elicit detectable structural changes in bone tissues.

## 5 Conclusion

This study investigated the role of Api in preventing the negative effects of LPS-induced inflammation on iWAT browning. In addition, the relationship between Api-induced browning and changes in the bone microenvironment was evaluated. The results showed that Api attenuates inflammation and promotes iWAT browning. In addition, Api promoted BMAT accumulation without adversely affecting trabecular or cortical bone parameters in LPS-injected mice treated with CL. Limitations of the present study include the lack of direct measurements of energy expenditure, the absence of functional assessments of bone strength, and the lack of long-term testing of Api supplementation, particularly in relation to bone metabolism. Additionally, the use of a non-obese model may limit the physiological relevance of our findings in the context of obesity. Future studies should include both male and female mice to assess potential sex-specific responses and enhance translational relevance. It is also recommended that dose-response experiments be conducted to better characterize the therapeutic range of Api. Despite these limitations, this study provides important insights into the therapeutic potential of Api in reducing inflammation and promoting adaptive thermogenesis in active inflammatory conditions. Nevertheless, further studies are required to assess the translational relevance and therapeutic potential of Api supplementation in humans.

## Data Availability

The original contributions presented in the study are included in the article/**Supplementary Material**. Further inquiries can be directed to the corresponding author.
