# Supplementary material for: Apigenin promotes remodeling of peripheral and skeletal adipocytes in response to β3-AR and TLR4 activation
Source: Front Endocrinol (Lausanne). 2025 Oct 17;16:1633584. doi: 10.3389/fendo.2025.1633584 (PMC12575153; doi:10.3389/fendo.2025.1633584)
Supplement: Supplementary file 1 [file DataSheet1.docx]

Supplementary Material

**Table S1. Primer sequences used for real-time PCR.**

| **Gene** | **Forward (5’-3’)** | **Reverse (5’-3’)** |
| --- | --- | --- |
| *36B4* | GGATCTGCTGCATCTGCTTG | GGCGACCTGGAAGTCCAACT |
| *Mcp-1* | AGGTCCCTGTCATGCTTCTG | GCTGCTGGTGATCCTCTTGT |
| *Cd11c* | CTGGATAGCCTTTCTTCTGCTG | GCACACTGTGTCCGAACTC |
| *Il-1β* | AAATACCTGTGGCCTTGGGC | CTTGGGATCCACACTCTCCAG |
| *Tnf-α* | GGCTGCCCCGACTACGT | ACTTTCTCCTGGTATGAGATAGCAAAT |
| *Ucp1* | AGGCTTCCAGTACCATTAGGT | CTGAGTGAGGCAAAGCTGATTT |
| *Cox8b* | CTCCCCCCTATCCTGCGGCTG | ACTATGGCTGAGATCCCCACA |
| *Elovl3* | GGGCCTCAAGCAAACCGTGTG | TGGCAGCCTTCATAGTGTAGT |
| *Dio2* | CAGTGTGGTGCACGTCTCCAATC | TGAACCAAAGTTGACCACCAG |
| *Cidea* | TGCTCTTCTGTATCGCCCAGT | GCCGTGTTAAGGAATCTGCTG |
| *Prdm16* | CAG CAC GGT GAA GCC ATT C | GCG TGC ATC CGC TTG TG |
| *Pgc1α* | CCCTGCCATTGTTAAGACC | TGCTGCTGTTCCTGTTTTC |
| *Cpt1b* | TCTGGGTGCTGGAGGTGGCTT | AACAGTGCTTGGCGGATGTGG |
| *Acadm* | AGACGAAGCCACGAAGTATGC | TCATCAGCTTCTCCACAGGGT |
| *Acadl* | TCTGGACTCCGGTTCTGCTTC | TCTGTCTTGCGATCAGCTCTT |
| *Fasn* | GGAGGTGGTGATAGCCGGTAT | TGGGTAATCCATAGAGCCCAG |
| *Acc* | TGGACAGACTGATCGCAGAGAAAG | TGGAGAGCCCCACACACA |
| *Dgat2* | CAGCATCCTCTCAGCCCTCC | CAGCCAGGTGAAGTAGAGCA |
| *PPARγ* | TGCGGAAGCCCTTTGGTGACT | ATGTCCTCGATGGGCTTCACGTTC |
| *Gapdh* | CATGGCCTTCCGTGTTCCTA | GCGGCACGTCAGATCCA |
| *aP2* | AGCATCATAACCCTAGATGGCG | CATAACACATTCCACCACCAGC |
| *Adipoq* | GAGATGCAGGTCTTCTTGGTC | GCTCTCCTTTCCTGCCAG |
| *Runx2* | AAATGCCTCCGCTGTTATGAA | GCTCCGGCCCACAAATCT |
| *Osx* | CCCTTCTCAAGCACCAATGG | AAGGGTGGGTAGTCATTTGCATA |
| *Alp* | ACACCAATGTAGCCAAGAATGTCA | GATTCGGGCAGCGGTTACT |

Supplementary Fig. 1


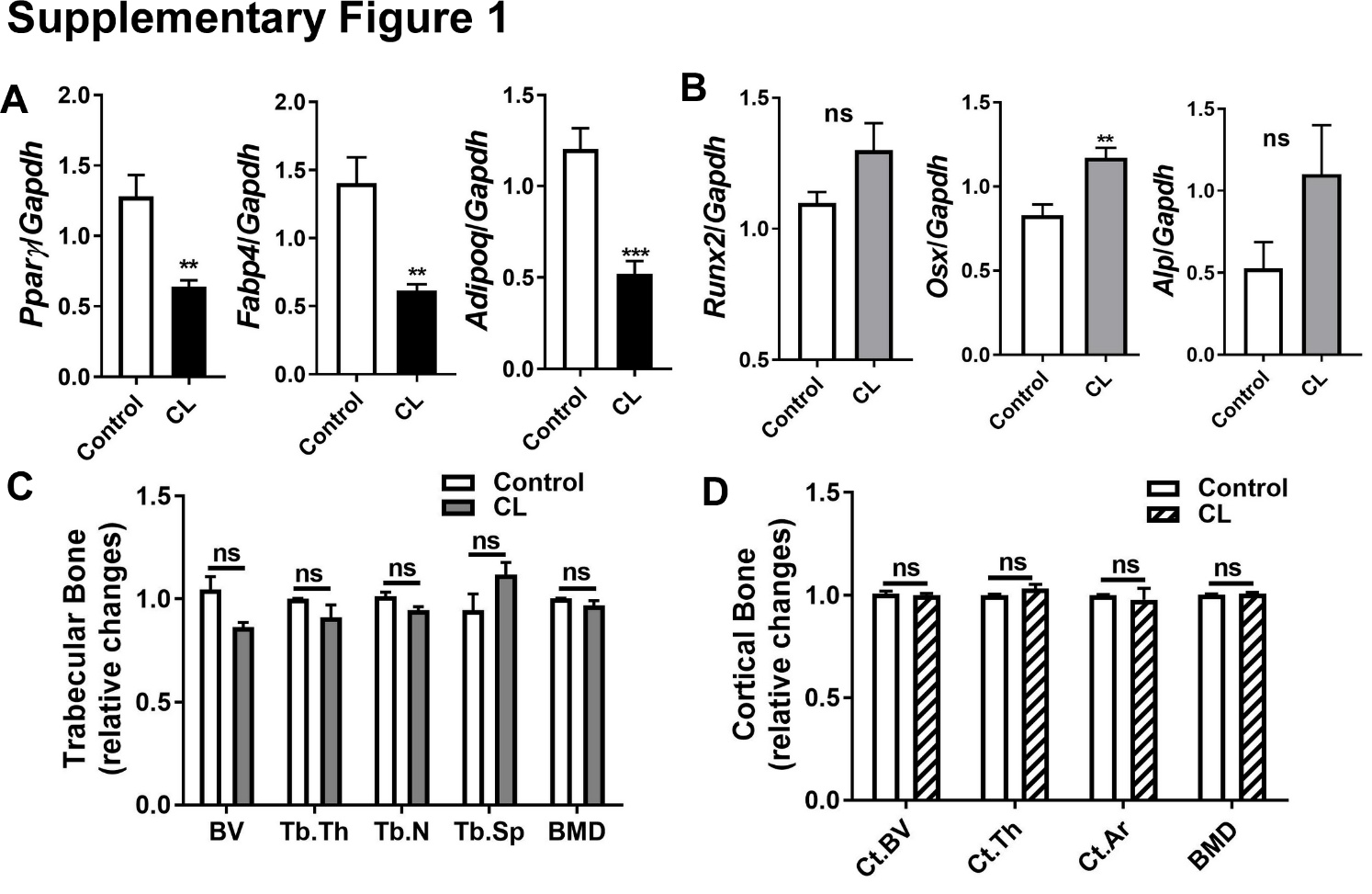


**Fig. S1. β3-adrenergic receptor activation suppresses adipogenic markers and modestly induces osteogenic markers in the bone microenvironment without affecting bone parameters.** C57BL/6 mice were administered either a vehicle or CL (1 mg/kg body weight) for 5 days. **A-B.** Relative gene expression analysis of adipogenic markers (A) in BM and osteogenic markers (B) in the tibia. **C-D.** µCT analysis of proximal trabecular (C) and cortical (D) tibial bone (n = 5). All data are presented as mean ± SEM. **P<0.01, ***P<0.001 by Student’s t-test.

Supplementary Fig. 2


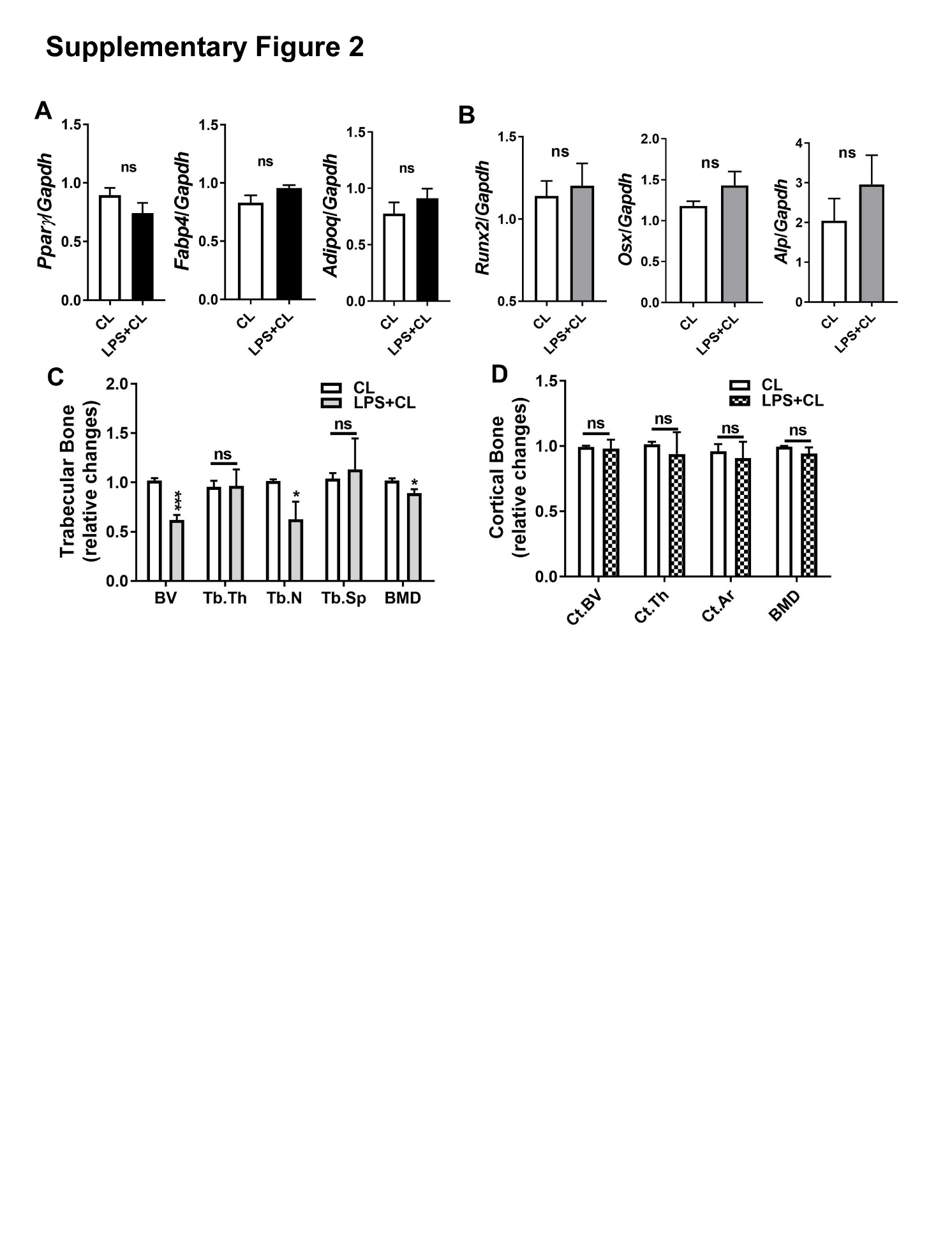


**Fig. S2. LPS administration has no effect on adipogenic and osteogenic markers in the bone microenvironment but negatively impacts trabecular bone parameters in mice undergoing adaptive thermogenesis.** C57BL/6 mice received either a vehicle or LPS (8 µg/mouse) every other day for 2 weeks, followed by CL (1 mg/ kg body weight) for the last 5 days. **A-B.** Relative gene expression analysis of adipogenic markers (A) in BM and osteogenic markers (B) in the tibia. **C-D.** µCT analysis of proximal trabecular (C) and cortical (D) tibial bone (n = 5). All data are presented as mean ± SEM. *P<0.05, ***P<0.001 by Student’s t-test.


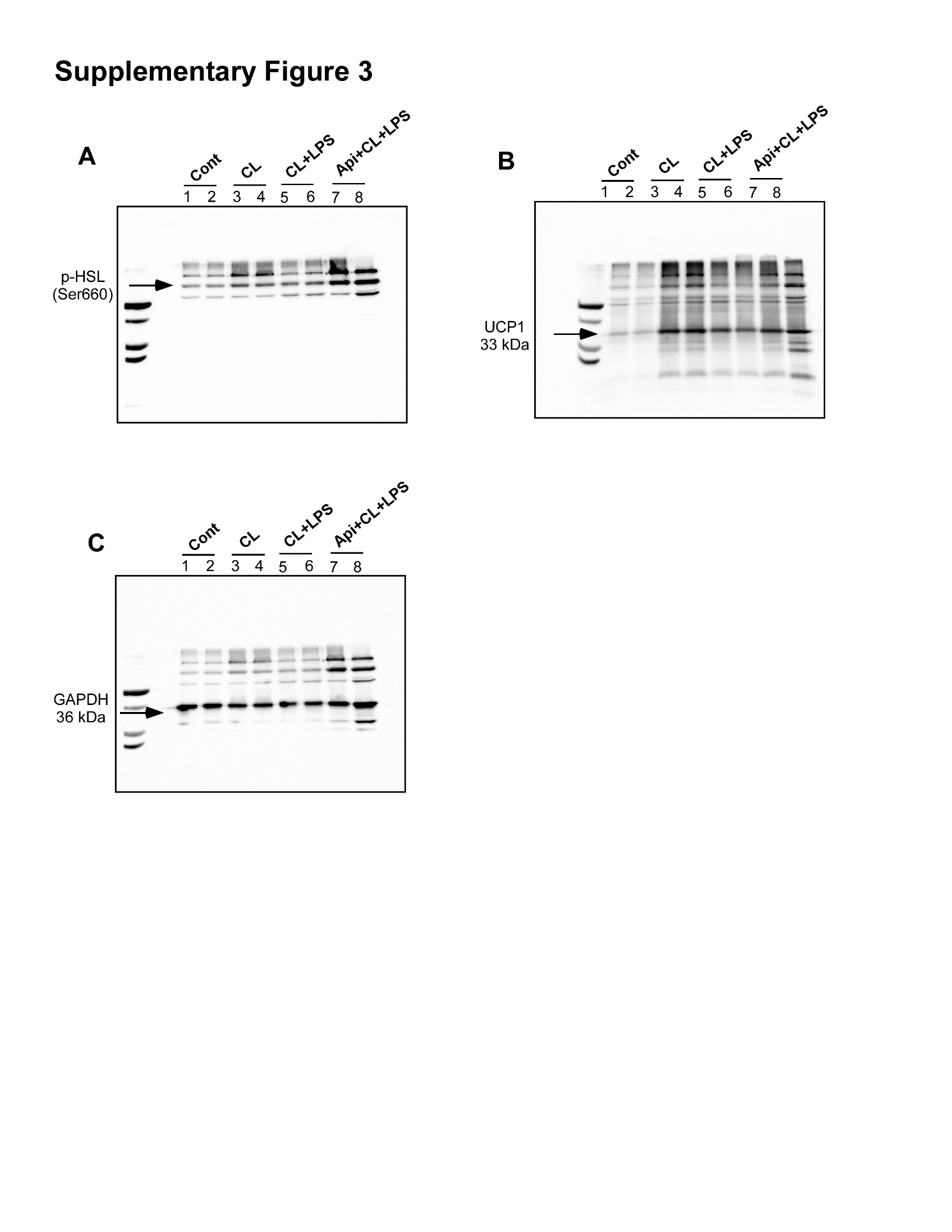
Supplementary Fig. 3

**Fig. S3. Full scans of Western blots from iWAT.** Full-length blots are presented for **A.** p-HSL (Ser660), at 81 and 83 kDa; **B.** UCP1, at 33kDa; **C.** GAPDH, at 36 kDa.

Supplementary Fig. 4


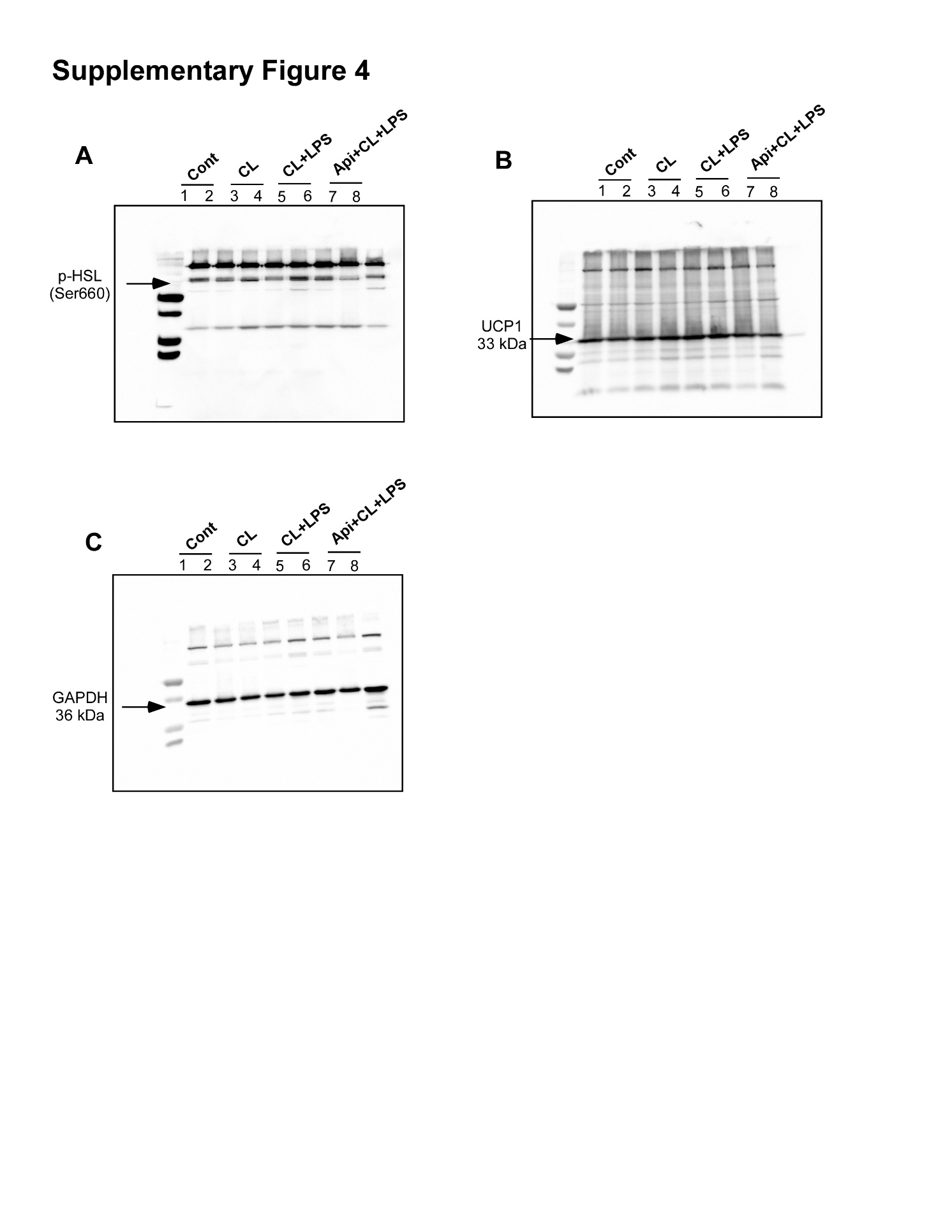


**Fig. S4. Full scans of Western blots from iBAT.** Full-length blots are presented for **A.** p-HSL (Ser660), at 81 and 83 kDa; **B.** UCP1, at 33kDa; **C.** GAPDH, at 36 kDa.

Supplementary Fig. 5


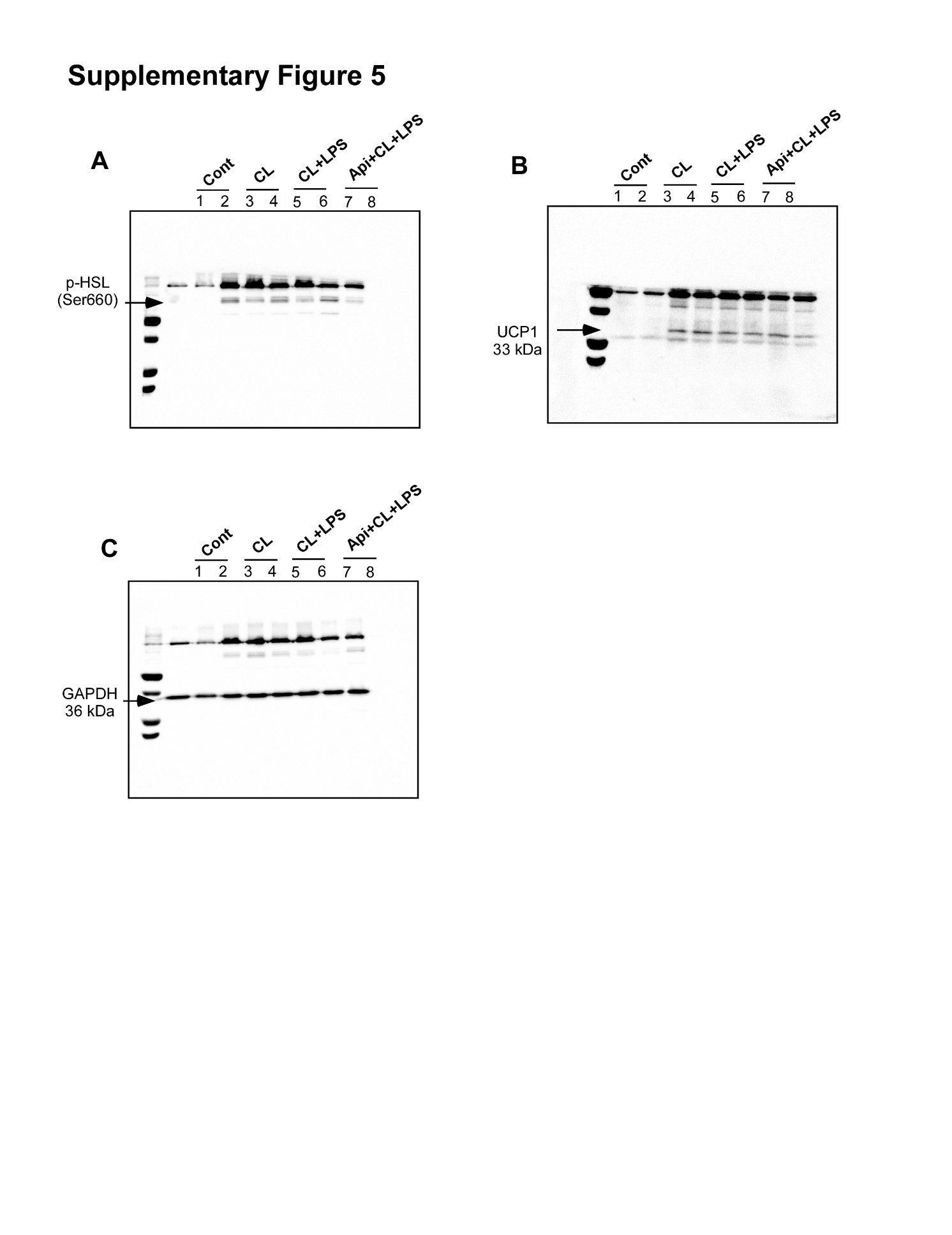


**Fig. S5. Full scans of Western blots from eWAT.** Full-length blots are presented for **A.** p-HSL (Ser660), at 81 and 83 kDa; **B.** UCP1, at 33kDa; **C.** GAPDH, at 36 kDa.

**
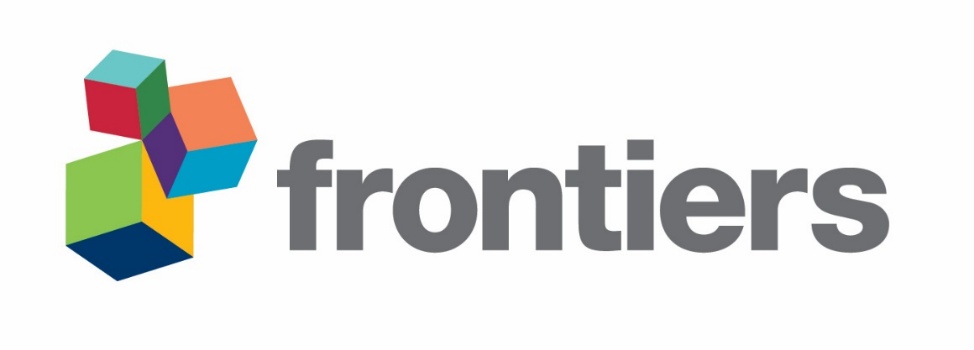
**
